# 

**Published:** 1862-08

**Authors:** 


### CONTENTS.

ORIGINAL ESSAYS AND COMMUNICATIONS.

Extracting Teeth. By DR. W. H. ATKINSON.... 341
The Extraction of Children's Teeth. By Dr W H. ATKINSON.... 344
Care of the Teeth. By. J. TAFT.... 347
Arsenic and its Compounds. By GEO. WATT.... 350
Success in the Dental Profession, No. 3. By C. C. DILLS, D. D. S.... 354
Metals and Alloys. By Dr. B. WOOD.... 357
Pivot Teeth. By W. H. SHADOAN.... 361

SELECTIONS.

Dentition and its Derangements. By J. JACOBI, M. D.... 363
Clinical Notes on Intemperance and its Prevention.... 369
The Ladies' Sanitary Association of London.... 375
Root of a Canine Tooth lodged in the thickness of the Lower Lip, simulating a Cancerous Tumor.... 376
Molar Tooth Lodged in the Tongue.... 376
On the Manna of Sinai, and the Manna of Syria.... 377
Deodoriziug the Breath and Cleansing the Teeth.... 380
Alveolar Abseess.... 381

EDITORIAL.

But One Kind of Dental Caries.... 382
More about Leeches.... 384
A New Dental Depot.... 385
Professional Signs and Advertisements.... 385
The New England Farmer.... 387

OBITUARY.

Dr. S. S. Gray, Memphis. Tenn.... 387
Carmi C. Franklin, New York City.... 388
Mr. Wakley, London. England.... 388

